# Village Practice

**Published:** 1917-11-24

**Authors:** 


### VILLAGE PRACTICE.

### An Autobiographical Fragment.

Two years' hospital appointments, concluding with the senior house surgeoncy at one of the chief London hospitals for children, were followed by three years of partnership in a high-class private practice. Then came a physical breakdown, necessitating a year or more of rest. After restoration, on a fateful morning towards the end of 1914, the Harley Street autocrat was delivering his verdict. Were I writing a popular novel the great man would, of course, be portrayed as sitting with easy dignity at his consulting-room table, and the description would necessarily involve reference to the strong, calm face, the steady steel-grey eyes, the finely moulded curve of the firm yet mobile mouth, etc. As I am, however, recording a matter of fact, I beg to state he was an English gentleman, strolling about his consulting-room, with one hand in his trousers pocket, whilst with the other he scratched his head meditatively, speaking the while thus: "It's like this, old chap; you've made a first-class recovery, but in order to keep well you'll have to chuck London and take things quietly in the country. I know you had ambitions, and I'm awfully sorry to turn you aside, but you can't again stand the strain of your old work. There will be handicaps and disappointments, of course, but there will also be compensations. You'll be able to get a bit of sport, and you'll have time for your books and for meditation. If you get near a hospital, your health will allow you to take an active part in it; but there is one difficulty in that connection. Small country hospitals are often jealously guarded preserves of, largely, a private nature, and the men on them are not always too anxious to share with others the local notoriety they enjoy. Keep a stiff upper lip. Do your own work in your own way. Widen your sphere, and for any sake don't let your sphere narrow you. Goodbye, old chap, let's know where you get to, and come up sometimes for a chat." A grip of the hand, and it was over. A good deal more had been said by the man at the top to his old student, but the gist of what is relevant to this paper was—"No climbing." Shortly after, a man in the wilds of Wiltshire, who had received five days' notice to join the R.A.M.C., for which he had volunteered, wanted someone to take his practice. Since things were as they were, I came, I saw, I succumbed!

"No climbing." No, not in an endeavour to scale the ladder of progress; but, after three years of it, I am deliberately of the opinion that a man in country practice must be struggling incessantly if he would keep on the ladder at all, otherwise he will be swept away into the morass of humbug and black magic that swirls around him.

An example. Before I had been here a month I received a visit from the traveller of a great firm of manufacturing chemists. Going round the surgery I asked him what some of the things were, as I had never heard of them. He advised me to throw them away and not to worry about them. "Some of them must have been here sixty years." My reply was that in that case it was to be hoped that a similar period of time had elapsed since the contents of the bottles had been used. On being asked to guide me in the choice of general paraphernalia, as I had hitherto been in the habit of writing prescriptions and knew nothing of dispensing, he asked, "Well, have you any special pill boxes?" As the special pill box was a mystery to me I could only reply, "I don't know; what are they?" Then on a combined search, and after rummaging about in various drawers, he found some diminutive receptacles that looked about equal to holding an ordinary 5-grain pill. These boxes, he explained, had been in use in country practice for many years, and had been made expressly for the repose of the special pill, which, it appeared, was prescribed at intervals for the patients who were informed that they needed it. In exchange for the special pill in its special box the patient handed the man of science half-a-crown, went home, took the extra-special, and, I hope, found he had his money's worth. "And what is the special pill?" I asked the traveller. "Oh, any old thing." On many occasions since my introduction to the "special pill" I have had to exclaim with Tennyson, "I falter where I firmly trod," but, before men and angels, I can, with a clear conscience, assert that in the matter of the special pill I am guiltless as yet. Even this modest boast I make with some inward qualms, for in country practice there is special need for the temporary victor to recall the exhortation, "Let him that thinketh he standeth, take heed lest he fall."

(*To be continued.*)

